# The Role of Muscular Fitness on Bone Mineral Content and Areal Bone Mineral Density in Youth With Type 1 Diabetes

**DOI:** 10.1210/clinem/dgaf328

**Published:** 2025-06-05

**Authors:** Jacinto Muñoz-Pardeza, Luis Gracia-Marco, José Francisco López-Gil, Ignacio Hormazábal-Aguayo, Nidia Huerta-Uribe, Andres Marmol-Perez, Yasmin Ezzatvar, Mikel Izquierdo, Antonio García-Hermoso

**Affiliations:** Navarrabiomed, Hospital Universitario de Navarra, Universidad Pública de Navarra (UPNA), IdiSNA, Pamplona 31008, Spain; Department of Physical Education and Sports, Faculty of Sport Sciences, Sport and Health University Research Institute (iMUDS), Granada 18007, Spain; CIBER for Physiopathology of Obesity and Nutrition (CIBEROBN), Carlos III Health Institute, Madrid 28029, Spain; Instituto de Investigación Biosanitaria, ibs. Granada, Granada 18012, Spain; School of Medicine, Universidad Espíritu Santo, Samborondón 0901952, Ecuador; Department of Communication and Education, Universidad Loyola Andalucía, Sevilla 41704, Spain; Navarrabiomed, Hospital Universitario de Navarra, Universidad Pública de Navarra (UPNA), IdiSNA, Pamplona 31008, Spain; Vicerrectoría de Investigación y Postgrado, Universidad de La Serena, La Serena 1700000, Chile; Navarrabiomed, Hospital Universitario de Navarra, Universidad Pública de Navarra (UPNA), IdiSNA, Pamplona 31008, Spain; Department of Physical Education and Sports, Faculty of Sport Sciences, Sport and Health University Research Institute (iMUDS), Granada 18007, Spain; Lifestyle Factors with Impact on Ageing and Overall Health (LAH) Research Group, Department of Nursing, University of València, Valencia 46010, Spain; Vicerrectoría de Investigación y Postgrado, Universidad de Los Lagos, Osorno 5290000, Chile; Navarrabiomed, Hospital Universitario de Navarra, Universidad Pública de Navarra (UPNA), IdiSNA, Pamplona 31008, Spain; CIBER of Frailty and Healthy Aging (CIBERFES), Carlos III Health Institute, Madrid 28029, Spain; Navarrabiomed, Hospital Universitario de Navarra, Universidad Pública de Navarra (UPNA), IdiSNA, Pamplona 31008, Spain

**Keywords:** bone health, childhood, dual-energy X-ray absorptiometry, insulin-dependent diabetes, resistance training

## Abstract

**Context:**

Type 1 diabetes in youth increases the risk of compromised bone health due to glycemic dysregulation. Muscular fitness may play a role in improving bone health during growth.

**Objective:**

This study aimed to investigate the association between muscular fitness and bone health in youth with type 1 diabetes.

**Methods:**

A total of 83 young individuals with type 1 diabetes (aged 6-18 years; 44.6% girls) from the Diactive-1 cohort study were followed for 2 years. Dual-energy x-ray absorptiometry whole-body scans were used to assess bone mineral content (BMC) and areal bone mineral density (aBMD) of the total body less head (TBLH), arms, legs, pelvis, and spine. Muscular fitness (handgrip strength, 1 repetition maximum, and muscle power) was assessed with a dynamometer and eGYM devices. Handgrip strength and TBLH bone parameters were age- and sex-standardized using the FitBack Project and BMD Childhood Study, respectively.

**Results:**

Linear mixed models showed longitudinal associations of handgrip strength with TBLH-BMC (unstandardized beta coefficient [*B*] = 17.18, 95% confidence interval [CI] 12.47-21.90) and TBLH-aBMD (*B* = 0.004, 95% CI 0.002-0.006); RM with TBLH-BMC (*B* = 20.09, 95% CI 10.88-29.31) and TBLH-aBMD (*B* = 0.007, 95% CI 0.004-0.011); and power with TBLH-BMC (*B* = 26.80, 95% CI: 17.31-36.28) and TBLH-aBMD (*B* = 0.009, 95% CI 0.005-0.012). Comparable results were observed across the other regions (*P* < .05). Additionally, analyses with standardized data confirmed the relationships of handgrip z-scores with TBLH-BMC z-scores (*B* = 0.19, 95% CI 0.08-0.30) and TBLH-aBMD z-scores (*B* = 0.350, 95% CI: 0.210-0.490).

**Conclusion:**

In pediatric patients with type 1 diabetes, higher muscular fitness could serve as a complementary therapeutic strategy to preserve or enhance bone health.

Type 1 diabetes is a chronic inflammatory metabolic disorder that causes autoimmune *B*-cell destruction, resulting in a loss of endogenous insulin production (1). The global incidence of type 1 diabetes is increasing at an annual rate of 3% to 4% (2), with a yearly incidence of 14.07 cases per 100 000 children and adolescents (3). Glycemic dysregulation contributes to a range of comorbidities, including poor bone health, which increases the risk of fractures (4). Consequently, current guidelines recommend bone health monitoring every 2 to 3 years via dual-energy x-ray absorptiometry (DXA) (5).

Youths with type 1 diabetes exhibit compromised bone strength (6), as evidenced by reduced areal bone mineral density (aBMD) and bone mineral content (BMC) (7), as well as alteration in other key parameters (8). These abnormalities have garnered significant attention due to their association with an elevated lifetime risk of fractures in individuals with type 1 diabetes compared to their healthy counterparts (9). Emerging evidence implicates glycemic dysregulation and chronic inflammation as pivotal factors influencing osteoblast-osteoclast dynamics and bone metabolism (10). Additionally, low levels of insulin-like growth factor 1 (IGF-1) (11), a key mediator of bone growth and turnover, further exacerbate these deficits.

Muscular fitness has emerged as a critical health marker during childhood and adolescence (12). Robust evidence demonstrates that higher levels of muscular fitness are associated with increased aBMD during developmental periods (13), with potential benefits extending into later life (14). Specifically, handgrip strength, a reliable indicator of muscular fitness, has been positively correlated with total body less head (TBLH) BMC in pediatric populations (15).

Youths with type 1 diabetes frequently present alterations in musculoskeletal function and reduced muscular fitness compared to healthy peers (16). This diminished physical capacity is not only associated with adverse musculoskeletal outcomes but also impacts broader health markers, including cardiometabolic health and telomere length (17, 18). In fact, the American Diabetes Association (19) recommends engaging in muscle-strengthening activities at least 3 times per week. These activities have been shown to improve glycemic control by reducing glycosylated hemoglobin (HbA1c) levels and insulin requirements (20).

Despite the increasing recognition of bone health as a concern in youth with type 1 diabetes, the longitudinal interaction between muscle fitness and bone outcomes in this population remains underexplored. Addressing this gap is crucial, given the unique challenges posed by glycemic dysregulation and musculoskeletal disorders. This study aimed to (a) analyze the associations of muscular fitness with aBMD and BMC outcomes in children and adolescents with type 1 diabetes from the Diactive cohort study and (b) assess these associations using reference values of handgrip z-scores and aBMD or BMC in TBLH. We hypothesized that increased muscle strength would be positively associated with positive outcomes on bone health parameters.

## Methods

### Study Design and Setting

The Diactive-1 cohort study is a longitudinal study evaluating children and adolescents with type 1 diabetes in Navarra (Spain) over 3 assessments within 2 years. Recruitment was performed at the Pediatric Diabetes Unit of the University Hospital of Navarra, between May 2021 and February 2022. Written informed consent was obtained from the parents or legal guardians, and the participants signed assent forms during the initial evaluation. Before each assessment, participants were contacted by telephone to schedule appointments and confirm their willingness to continue. Assessments, including baseline and 2 annual follow-ups, were performed in a specialized youth-friendly exercise laboratory at the Navarrabiomed Research Center, under the supervision of a multidisciplinary team. Only specialized technicians who were blinded to the context of the participants handled the measurement instruments. The sessions lasted approximately 90 minutes, were scheduled early in the morning, and guaranteed rigorous glycemic control (21). The Research Ethics Committee on Medicines approved the project at the University Hospital of Navarra (PI_2021/32) following the ethical guidelines of the Declaration of Helsinki (revised version 2013). As shown in Supplementary Table S1 (22), this study was reported according to the Strengthening the Reporting of OBservational Studies in Epidemiology (STROBE) checklist.

### Participants

This study included children and adolescents aged 6 to 18 years who had been diagnosed with type 1 diabetes for more than 6 months. The exclusion criteria included comorbidities that restricted participation in physical activities, such as cardiovascular disease or severe obesity, being in the honeymoon phase (defined as insulin requirements ≤0.5 U/kg/day and HbA1c ≤ 6%) (23), or a lack of sufficient understanding of the Spanish language. However, none of the participants were excluded from the study. Of the 183 patients assessed in the Pediatric Endocrinology Unit, 143 met the eligibility criteria and 83 consented to participate, leading to a 58.04% participation rate. Participation was entirely voluntary, and those who declined to participate were not required to justify their decision. Therefore, no information was collected on the reasons for nonparticipation. In addition, the sample sizes varied slightly for some variables because of missing data for random reasons. However, this was mainly because some participants were unable to take specific tests or were apprehensive about undergoing DXA scans.

### Variables

#### Anthropometric parameters and peak height velocity

Standing height was measured with a SECA 123 stadiometer with an accuracy of 0.1 cm (Hamburg, Germany), whereas sitting height was assessed using a 42-centimeter-high wooden box. The participants stood or sat with themselves together, with their backs straight and heads aligned in the Frankfurt horizontal plane, ensuring complete contact with the stadiometer column. Body weight was measured with the participants dressed in light clothing, with nothing in their pockets, and they were asked to release all the air at the time of measurement; for this purpose, an SECA 869 electronic scale with an accuracy of 0.1 kg was used. Body mass index (BMI) was calculated as body mass divided by height squared (kg/m^2^). Age- and sex-specific BMI z-scores were determined via international reference data (24).

Somatic maturation was assessed based on years estimated from the peak height velocity (PHV), a key marker of pubertal growth and development in children and adolescents. Moore's equation, which incorporates height, sitting height, weight, sex, and age, was used to calculate this parameter (25). To analyze the trajectories of the dependent variables along the maturational stages, participants were classified as prepubertal (≤ −1 year since PHV), peripubertal (−1 < year since PHV < 1), or postpubertal (≥1 year since PHV) (26).

#### Assessment of parameters related to diabetes and calcium intake

In accordance with patient data protection regulations, medical records were reviewed by a pediatric endocrinologist to extract information on disease duration and HbA1c levels. To ensure data accuracy, a second qualified investigator independently verified the extracted data. For patients without a recent blood test (within 3 months prior to evaluation), testing was requested within a few days of assessment. HbA1c levels were analyzed at the central laboratory of the University Hospital of Navarra (Spain). In addition, the daily insulin dose (U/kg) was extracted from the software associated with the FreeStyle® continuous glucose measurement devices (Abbott Diabetes Care, Chicago, IL, USA) and MiniMedTM devices (Medtronic, Minneapolis, MN, USA) over 9 days. These devices were not supplied as part of the study but rather reflected the diabetes management systems already in use by the participants.

Daily calcium intake (in milligrams) was estimated using a validated specific food frequency questionnaire (27).

#### Total physical activity

According to previous research, total physical activity can positively influence different bone parameters in children and adolescents with type 1 diabetes (28). Therefore, total physical activity was quantified using the GENEActive triaxial accelerometer (ActiveInsights, Huntingdon, United Kingdom) worn on the wrist of the nondominant arm. The prerecording configuration was based on 85.7 Hz for 9 consecutive days (29), beginning at 08:00:00 Pm on the evaluation day. The first night was allocated for calibration, while the subsequent days were synchronized with the continuous glucose monitoring recordings. “GENEActivPCSoftware” (version 3.3) was used to collect accelerometer data, and the *GGIR* package was used to process the information (30).

A minimum of 7 days of complete daily movement data, including at least one weekend day, were needed to ensure robust analysis. The device was required to be worn for the entire 24-hour period, including during sleep time. In addition, to understand the daily movement behavior of the participants, accelerometric recordings were complemented with a diary. In this diary, participants documented when and why they removed their wrist devices. Total physical activity was determined by summing the following variables, which were determined according to cutoff points established for children and adolescents (31, 32): light activity (for children: 56.3-191.6 milli-gravity units [m*g*]; for adolescents: 50-150 m*g*), moderate activity (for children: 191.6-695.8 m*g*; for adolescents: 150-500 m*g*), and vigorous activity (for children: >695.8 m*g*; for adolescents: >500 m*g*).

#### Bone mineral content and bone mineral density (dependent variables)

Children and adolescents with type 1 diabetes were evaluated using a single DXA scanner (DXA Lunar iDXA, GE HealthCare^©^, Chicago, USA) and analyzed with enCORE v.18 software. Following the International Society for Clinical Densitometry guidelines, the device was calibrated daily before the measurements, during which the participants were instructed to remain motionless in the supine position (33).

Given that data collection for this cohort did not focus on bone-specific parameters as primary outcomes, total body scans were performed to determine the aBMD (g/cm^2^) and BMC (g) of the TBLH, arms, legs, pelvis, and spine.

Additionally, age-, sex-, and race/ethnicity-specific z-scores for aBMD and BMC at TBLH were calculated via the international Lambda, Mu, and Sigma reference data of the Bone Mineral Density in Childhood Study (34).

#### Muscular fitness (independent variable)

Muscular fitness was assessed using different methods. Handgrip strength was measured with a Takei III Smedley Type 297 Digital Dynamometer (Takei Scientific Instruments, Niigata, Japan). The measurement protocol was based on measuring the width of the hand to adjust the device individually. Two repetitions were performed with each arm alternately, and an average was calculated between the highest scores of the right and left arms. In addition, eGYM Smart Strength machines (eGym® GmbH, Munich, Germany) were used to evaluate the force executed in one repetition maximum (1RM) and the mean muscle power exerted in 10 repetitions for hip press, vertical press, quadriceps curl, and vertical rowing. To minimize muscle fatigue, upper limb (push and pull) and lower limb (hip and knee dominant) exercises were alternated during the assessment. For the resulting data for each body action, Z values were calculated and summed to obtain both RM and overall muscle power.

In addition, guidelines recommend handgrip strength as a valuable measure of muscular fitness (35). Therefore, we decided to standardize our handgrip data with the reference population, and z-scores were calculated with percentiles according to age and sex provided by the FitBack project (36). In the final stage of data processing, youths with a handgrip strength ≤20th percentile were classified as “insufficient,” as shown in previous studies (37).

### Statistical Methods

The normality of the variable distributions was evaluated via density diagrams and quantile-quantile plots, complemented with the Shapiro-Wilk test for statistical verification. Descriptive statistics are presented as follows: categorical variables are summarized as frequencies and proportions (%), whereas continuous variables are expressed as means with SD when their distribution is normal or as medians accompanied by interquartile ranges (IQRs) for data without a normal distribution. Changes between baseline and second follow-up measurements were analyzed using paired *t* tests for continuous variables with normal distributions. In contrast, the Wilcoxon test was applied for the non-normally distributed data. Differences in bone health parameters were investigated by sex, age group, and pubertal stage (classified as peripubertal or postpubertal), and the mean differences were also estimated. Furthermore, the baseline characteristics of the participants who completed the study were compared with those who voluntarily withdrew, employing a *t* test for independent samples or Mann-Whitney *U* test, as shown in Supplementary Table S2 (22).

Generalized linear mixed models (GLMM) were used to examine the associations between muscular fitness and both BMC and aBMD across different body sections by estimating unstandardized beta coefficients (*B*) and their corresponding 95% CIs, incorporating key variables as fixed effects in 2 different models: timing of measurements, HbA1c, daily insulin dose, diabetes duration, PHV, and BMI (*Model 1*), as well as the above plus total physical activity and calcium intake (*Model 2*). In the 2 previous models, subject identification was incorporated as random effect to control for intraindividual variability over time (38). Sensitivity analyses were performed by inspecting interactions to consider the influence of time between measurements and possible hormonal variations between maturation stages, which could affect the direction, slope, or significance of the observed associations.

To identify the minimum sufficient set of adjustments for associations between muscular fitness and aBMD or BMC, we constructed a directed acyclic graph (DAG) using the online tool DAGitty (39), as shown in Supplementary Fig. S1 (22). Additionally, to provide a clinical perspective, the relationships between z-handgrip strength, Z-BMC, and Z-aBMD for TBLH, including the interaction of assessment time points, were analyzed. Finally, differences across the 3 assessments were compared between individuals with handgrip strength ≤20th percentile and those above this threshold (37).

For this purpose, all data analyses were performed via R language (4.4.1; R Core Team, Vienna, Austria) with RStudio software (2024.09.1; RStudio, Boston, MA, USA). The models were fitted via the “*lmer*” function of the *lme4* package, ensuring robust and accurate inference about the longitudinal relationships of interest (40). After evaluation of the assumptions underlying each model (eg, homoscedasticity and normality of the residuals), the analyses were rerun employing the “*rlmer*” function of the *robustlmm* package, providing a more reliable estimation framework against the presence of outliers, heteroscedasticity, and influential observations that could bias the results (41). Additionally, the conditional coefficient of determination (*R*_c_^2^) was computed via the *MuMIn* package to determine the total variance of fixed and random effects (42) explained. The intraclass correlation coefficient (ICC) was calculated. The reason for data loss was studied using the *naniar* package, and it was considered that this was due to random reasons (43). Likewise, it was considered not to impute missing data because the robustness of the applied models allowed the inclusion of all values without significant biases.

The statistical framework was designed to address 3 questions: (a) the trajectory of bone parameters over time; (b) the relationships between different levels of muscular fitness and bone health parameters; and (c) how this relationship fluctuates at different points in time and between individuals. Statistical significance was set at *P* < .05 for all outcomes.

## Results

### Main Characteristics

The study cohort consisted of 83 participants with a mean age of 13.7 ± 2.8 years, a mean diabetes duration of 5.8 ± 3.5 years, and an average HbA1c level of 7.4 ± 0.8%. Detailed descriptive characteristics of the participants at each measurement point and comparisons between the baseline and second year of follow-up are presented in Table 1. Significant differences (*P* < .05) were found for all variables, except BMI z-score (*P* = .709), handgrip strength z-score (*P* = .077), total RM (*P* = .258), total power (*P* = .417), TBLH-BMC z-score (*P* = .675), and TBLH-aBMD z-score (*P* = .570). For the BMC and aBMD parameters, data losses of 0% during follow-up 1 and 1.6% during follow-up 2 were observed.

**Table 1.** Baseline and follow-up participant characteristics in the Diactive-1 Cohort Study

Variables	At baseline n = 83	At 1 year follow-up n = 64	At 2 years follow-up n = 62	*P*
Age, years	13 (4)*^b^*	14 (4)*^b^*	15 (4)*^b^*	**<.001**
Diabetes duration, years	4 (5)*^b^*	5 (5)*^b^*	6 (5)*^b^*	**<.001**
Sex				
Boys (%)	55	52	52	.774
Girls (%)	45	48	48	.774
Anthropometric				
Body height, m	1.60 (0.20)*^b^*	1.60 (0.17)*^b^*	1.60 (0.14)*^b^*	**<.001**
Body weight, kg	51.50 ± 16.99*^a^*	55.14 ± 16.19*^a^*	57.87 ± 15.91*^a^*	**<.001**
Body mass index, kg/m^2^	19.39 (4.64)*^b^*	20.71 (4.33)*^b^*	20.90 (5.24)*^b^*	**<.001**
Body mass index, z-score	0.6 ± 0.9*^a^*	0.6 ± 0.9*^a^*	0.5 ± 1.0*^a^*	.709
Peak height velocity score	−0.3 ± 1.9*^a^*	0.3 ± 1.9*^a^*	0.9 ± 1.8*^a^*	**<.001**
Diabetes-related assessment				
HbA1c, mmol/mol	80 ± 16*^a^*	57 ± 12*^a^*	61 ± 14*^a^*	**.002**
HbA1c, %	7.4 ± 0.8*^a^*	7.4 ± 1.1*^a^*	7.7 ± 1.2*^a^*	**.002**
Daily insulin dose, U/kg	0.69 (0.29)*^b^*	0.77 (0.32)*^b^*	0.85 (0.29)*^b^*	**.025**
Physical activity				
Total PA, min	332 ± 81*^a^*	328 ± 83*^a^*	303 ± 80*^a^*	**<.001**
Muscular fitness				
Handgrip strength, z-score	−0.5 (0.9)*^b^*	−0.5 (1.1)*^b^*	−0.5 (1.0)*^b^*	.077
Handgrip strength, kg	20.6 (11.1)*^b^*	20.8 (12.1)*^b^*	23.5 (12.5)*^b^*	**<.001**
Total RM, score	−0.4 (4.0)*^b^*	−0.69 (4.3)*^b^*	−0.2 (4.9)*^b^*	.258
Total power, score	−0.5 (3.9)*^b^*	−0.9 (3.9)*^b^*	−0.5 (4.9)*^b^*	.417
Bone health parameters				
*Bone mineral content (BMC)*				
TBLH, z-score	1.0 ± 1.1*^a^*	1.1 ± 1.1*^a^*	1.0 ± 1.0*^a^*	.675
TBLH, g	1510.32 ± 554.00 *^a^*	1641.59 ± 522.31*^a^*	1744.12 ± 512.93*^a^*	**<.001**
Arms, g	232.62 ± 86.76*^a^*	257.99 ± 86.09*^a^*	276.09 ± 89.86*^a^*	**<.001**
Legs, g	730.44 ± 260.58*^a^*	788.64 ± 242.69*^a^*	838.03 ± 236.47*^a^*	**<.001**
Pelvis, g	239.15 (172.12)*^b^*	265.74 (156.77)*^b^*	280.18 (128.73)*^b^*	**<.001**
Spine, g	127.10 (82.90)*^b^*	138.24 (73.58)*^b^*	149.42 (77.97)*^b^*	**<.001**
*Areal bone mineral density (aBMD)*				
TBLH, z-score	0.8 (1.6)*^b^*	0.9 (1.6)*^b^*	0.8 (1.6)*^b^*	.570
TBLH, g/cm^2^	0.908 ± 0.170*^a^*	0.951 ± 0.164*^a^*	0.977 ± 0.162*^a^*	**<.001**
Arms, g/cm^2^	0.704 ± 0.130*^a^*	0.737 ± 0.133*^a^*	0.746 ± 0.138*^a^*	**<.001**
Legs, g/cm^2^	1.070 ± 0.200*^a^*	1.124 ± 0.196*^a^*	1.164 ± 0.193*^a^*	**<.001**
Pelvis, g/cm^2^	0.903 (0.309)*^b^*	0.947 (0.272)*^b^*	0.972 (0.260)*^b^*	**<.001**
Spine, g/cm^2^	0.872 (0.303)*^b^*	0.905 (0.314)*^b^*	0.946 (0.311)*^b^*	**<.001**

*P* value determined by Wilcoxon or paired *t* test for differences between baseline and second-year follow-up.

Bold (*P* < .05) implies difference in means.

Abbreviations: HbA1c, glycosylated hemoglobin; PA, physical activity; RM, maximum repetition; TBLH, total body less head.

^*a*^Normal variables: mean ± SD.

^*b*^Nonnormal variables: median (interquartile range).

As illustrated in Supplementary Fig. S2 (22), 64 participants completed the first year of follow-up (23% voluntary dropout) and 62 completed the second year (26% voluntary dropout). No significant differences were identified between the participants who completed the study and those who dropped out in any variable but the handgrip strength z-score (*P* = .022) (Supplementary Table S2) (22).

### Longitudinal Associations

The associations between muscular fitness and BMC across various body regions are presented in Table 2. In *Model 1*, significant positive relationships were observed between handgrip strength (*B* = 16.185, 95% CI 11.581-20.788), RM (*B* = 20.232, 95% CI 11.312-29.151), and power (*B* = 27.907, 95% CI 18.818-36.995) with TBLH-BMC. Comparable significant results were identified across the other body regions analyzed in this model. Similarly, in *Model 2*, these associations remained robust and significant for most relationships (*P* < .05).

**Table 2.** Multiple linear regressions for the associations by 2-year longitudinal linear mixed model analysis between different parameters of muscular fitness and bone mineral content in different body sections

	*Total body less head*							
	*Model 1*				*Model 2*			
*Main predictors*	*B* (SE)	95% CI	*R_c_* ^2^	ICC	*B* (SE)	95% CI	*R_c_* ^2^	ICC
Handgrip	**16.185** (2.349)	11.581 to 20.788	0.98	0.91	**16.713** (2.757)	11.309 to 22.117	0.98	0.92
RM	**20.232** (4.551)	11.312 to 29.151	0.98	0.92	**19.535** (4.712)	10.299 to 28.771	0.98	0.92
Power	**27.907** (4.637)	18.818 to 36.995	0.98	0.92	**28.179** (4.950)	18.475 to 37.882	0.98	0.90

	*Arms*							
	*Model 1*				*Model 2*			
*Main predictors*	*B* (SE)	95% CI	*R_c_^2^*	ICC	*B* (SE)	95% CI	*R_c_^2^*	ICC
Handgrip	**3.552** (0.439)	2.691 to 4.412	0.97	0.84	**3.395** (0.512)	2.390 to 4.399	0.97	0.83
RM	**3.427** (0.895)	1.673 to 5.182	0.97	0.87	**3.279** (0.936)	1.444 to 5.114	0.97	0.87
Power	**4.358** (0.919)	2.556 to 6.160	0.97	0.87	**4.257** (0.958)	2.378 to 6.136	0.97	0.88

	*Legs*							
	*Model 1*				*Model 2*			
*Main predictors*	*Β* (SE)	95% CI	*R_c_^2^*	ICC	*B* (SE)	95% CI	*R_c_^2^*	ICC
Handgrip	**7.679** (1.356)	5.021 to 10.336	0.97	0.91	**8.192** (1.601)	5.053 to 11.330	0.97	0.90
RM	**9.566** (2.476)	4.712 to 14.421	0.98	0.92	**10.116** (2.670)	4.882 to 15.350	0.97	0.92
Power	**14.029** (2.433)	8.559 to 18.593	0.98	0.93	**14.881** (2.669)	9.649 to 20.113	0.98	0.92

	*Pelvis*							
	*Model 1*				*Model 2*			
*Main predictors*	*B* (SE)	95% CI	*R_c_^2^*	ICC	*B* (SE)	95% CI	*R_c_^2^*	ICC
Handgrip	**3.418** (0.551)	2.338 to 4.498	0.96	0.81	**3.608** (0.628)	2.376 to 4.840	0.97	0.83
RM	**5.141** (1.029)	3.124 to 7.158	0.97	0.86	**5.031** (1.069)	2.936 to 7.127	0.97	0.79
Power	**6.214** (1.057)	4.142 to 8.286	0.97	0.88	**6.344** (1.118)	4.152 to 8.537	0.97	0.88

	*Spine*							
	*Model 1*				*Model 2*			
*Main predictors*	*B* (SE)	95% CI	*R_c_^2^*	ICC	*B* (SE)	95% CI	*R_c_^2^*	ICC
Handgrip	**0.775** (0.279)	0.227 to 1.323	0.97	0.91	**0.695** (0.326)	0.055 to 1.336	0.97	0.91
RM	**1.284** (0.525)	0.255 to 2.313	0.97	0.91	**1.160** (0.552)	0.076 to 2.244	0.97	0.91
Power	**1.627** (0.543)	0.562 to 2.691	0.97	0.91	**1.555** (0.576)	0.426 to 2.685	0.97	0.91

*Model 1:* Adjusted for year of assessment, glycosylated hemoglobin, daily insulin dose, disease duration, peak height velocity, body mass index and the random variable [participant identification]. The number of observations was 185 for handgrip and 179 for RM and power.

*Model 2:* Adjusted by the same variables as in *Model 1* + total physical activity and calcium intake. The number of observations was for handgrip and for RM and power. The number of observations was 170 for handgrip and 164 for RM and power.

Bold (*P* < .05) implies difference in means.

Abbreviations: *B*, unstandardized beta coefficient; ICC, intraclass correlation coefficient; *R_c_^2^*, conditional coefficient of determination; RM, maximum repetition; SE, standard error.

Table 3 summarizes the associations between muscular fitness and aBMD across various body regions. In *Model 1*, significant positive relationships were observed between handgrip strength (*B* = 0.004, 95% CI 0.002-0.006), RM (*B* = 0.007, 95% CI 0.004-0.011), and power (*B* = 0.009, 95% CI 0.006-0.013) with aBMD, not only for TBLH but also for the other body regions analyzed (*P* < .05). *Model 2* yielded similar results, with statistically significant associations persisting for almost all parameters examined (*P* < .05).

**Table 3.** Associations by 2-year longitudinal linear mixed model analysis between different parameters of muscular fitness and areal bone mineral density in different body sections

	*Total body less head*							
	*Model 1*				*Model 2*			
*Main predictors*	*B* (SE)	95% CI	*R_c_^2^*	ICC	*B* (SE)	95% CI	*R_c_^2^*	ICC
Handgrip	**0.004** (0.000)	0.002 to 0.006	0.97	0.80	**0.004** (0.001)	0.001 to 0.006	0.97	0.78
RM	**0.007** (0.001)	0.004 to 0.011	0.97	0.79	**0.007** (0.001)	0.003 to 0.011	0.97	0.78
Power	**0.009** (0.001)	0.006 to 0.013	0.97	0.78	**0.009** (0.001)	0.005 to 0.013	0.97	0.77

	*Arms*							
	*Model 1*				*Model 2*			
*Main predictors*	*B* (SE)	95% CI	*R_c_^2^*	ICC	*B* (SE)	95% CI	*R_c_^2^*	ICC
Handgrip	**0.002** (0.001)	0.001 to 0.005	0.91	0.64	**0.002** (0.001)	0.000 to 0.005	0.91	0.66
RM	**0.006** (0.001)	0.002 to 0.010	0.92	0.68	**0.006** (0.002)	0.002 to 0.010	0.92	0.70
Power	**0.006** (0.001)	0.002 to 0.009	0.92	0.66	**0.006** (0.002)	0.001 to 0.010	0.92	0.69

	*Legs*							
	*Model 1*				*Model 2*			
*Main predictors*	*B* (SE)	95% CI	*R_c_^2^*	ICC	*B* (SE)	95% CI	*R_c_^2^*	ICC
Handgrip	**0.007** (0.001)	0.004 to 0.009	0.97	0.88	**0.005** (0.001)	0.003 to 0.008	0.96	0.88
RM	**0.009** (0.002)	0.005 to 0.014	0.97	0.88	**0.006** (0.002)	0.004 to 0.013	0.97	0.88
Power	**0.006** (0.001)	0.007 to 0.016	0.97	0.89	**0.006** (0.002)	0.006 to 0.015	0.97	0.89

	*Pelvis*							
	*Model 1*				*Model 2*			
*Main predictors*	*B* (SE)	95% CI	*R_c_^2^*	ICC	*B* (SE)	95% CI	*R_c_^2^*	ICC
Handgrip	**0.005** (0.001)	0.002 to 0.008	0.96	0.88	**0.006** (0.001)	0.003 to 0.009	0.96	0.89
RM	**0.009** (0.002)	0.004 to 0.014	0.96	0.89	**0.009** (0.002)	0.004 to 0.014	0.96	0.88
Power	**0.013** (0.002)	0.008 to 0.018	0.96	0.90	**0.014** (0.002)	0.008 to 0.019	0.96	0.90

	*Spine*							
	*Model 1*				*Model 2*			
*Main predictors*	*B* (SE)	95% CI	*R_c_^2^*	ICC	*B* (SE)	95% CI	*R_c_^2^*	ICC
Handgrip	**0.003** (0.001)	0.001 to 0.005	0.97	0.90	0.002 (0.001)	−0.000 to 0.004	0.97	0.89
RM	**0.005** (0.002)	0.001 to 0.009	0.97	0.89	**0.005** (0.002)	0.001 to 0.009	0.97	0.89
Power	**0.007** (0.002)	0.003 to 0.011	0.97	0.90	**0.007** (0.002)	0.003 to 0.011	0.97	0.90

*Model 1*: Adjusted for year of assessment, glycosylated hemoglobin, daily insulin dose, disease duration, peak height velocity, body mass index and the random variable [participant identification]. The number of observations was 185 for handgrip and 179 for RM and power.

*Model 2*: Adjusted by the same variables as in *Model 1* + total physical activity and calcium intake. The number of observations was for handgrip and for RM and power. The number of observations was 170 for handgrip and 164 for RM and power.

Bold (*P* < .05) implies difference in means.

Abbreviations: *B*, unstandardized beta coefficient; ICC, intraclass correlation coefficient; R_c_^2^, conditional coefficient of determination; RM, maximum repetition; SE, standard error.

**Figure 1.**
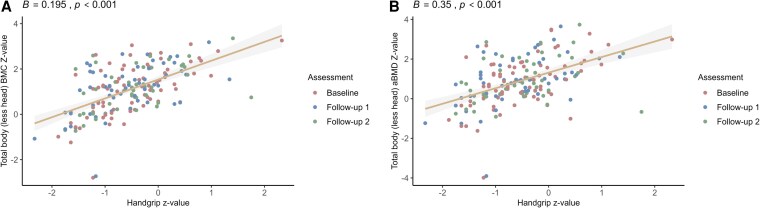
Results of the 2-year longitudinal multiple linear mixed model analysis for the associations of handgrip strength standardized z-scores based on reference percentiles with age-, sex- and race-specific bone mineral content (BMC) z-score (A) or areal bone mineral density (aBMD) z-scores (B).

Moreover, the generalized linear mixed model results assessing the relationship between the standardized z-score of handgrip strength and the z-scores for TBLH-BMC and aBMD are provided in Supplementary Table S3 (22) and illustrated in Fig. 1. Significant associations were identified for both the BMC z-score (*B* = 0.194, 95% CI 0.088-0.300) and the aBMD z-score (*B* = 0.350, 95% CI 0.210-0.490). Additionally, Supplementary Fig. S3 (22) highlights that being above the 20th percentile in the handgrip z-score was associated with a significantly higher BMC z-score and aBMD z-score than below the 20th percentile (*P* < .001).

### Potential Interactions

Supplementary Table S4 (22) shows no significant interactions of evaluation time or maturation on the associations between muscular fitness (RM or power) and BMC across body regions (*P* > .05). However, handgrip strength showed significant interactions with BMC in certain areas, particularly between the follow-up and baseline assessments and across maturation stages (*P* < .05). Similarly, Supplementary Table S5 (22) highlights interactions between handgrip strength and aBMD in the TBLH, legs, and pelvis (*P* < .05). Supplementary Fig. S4 (22) confirms the consistency of associations between handgrip strength and bone outcomes (BMC or aBMD z-scores) over time (*P* > .80).

## Discussion

### Main Findings

This study highlights that, among children and adolescents with type 1 diabetes, increased muscular fitness is strongly associated with positive bone health outcomes, specifically with higher BMC and aBMD across various skeletal regions. These associations were observed across different dimensions of muscle fitness, with muscle power emerging as a particularly important factor. Notably, higher standardized handgrip strength scores, aligned with updated reference values, were consistently linked to greater sex-, age-, and race/ethnicity-standardized BMC and aBMD in TBLH, even showing that the relationships were stable longitudinally. Moreover, individuals with muscular fitness deficits exhibited significantly poorer bone health compared to those without such deficits. The findings support current clinical guidelines recommending regular monitoring of bone health in youth with type 1 diabetes via DXA (5). They also reinforce existing recommendations advocating at least 3 days per week of muscle-strengthening activities (19), as a critical component of care for this population.

To the best of our knowledge, this study contributes to the limited literature exploring the relationship between muscular fitness and bone health in youth with type 1 diabetes. For instance, Maratova et al (44) compared the BMD and muscle function of young individuals with type 1 diabetes to those of a reference population, whereas Dongare-Bhor et al (45) examined the correlation of handgrip strength with aBMD and BMC, reporting findings consistent with ours. Although the above results are consistent with ours, one of the strengths is that we included maturational stage, an essential confounder, as an adjusted covariate and as an interaction factor to explore possible variations in the associations, allowing us to indirectly account for possible hormonal influences on muscle-bone growth at all developmental stages. In regions such as TBLH, our results suggest an increased mechanical sensitivity of bone to muscle forces during prepuberty, prior to the onset of possible dominant pubertal hormonal influences that could progressively modulate bone accretion and attenuate the relative contribution of muscle strength. Furthermore, one randomized clinical trial implemented a 9-month strength training program of 180 minutes per week, demonstrating significant effects on impaired aBMD (46) in youth with type 1 diabetes. This finding aligns with the positive associations observed in our study. Notably, the trial incorporated both strength training and competitive sports rather than isolated, systematic resistance training, and did not examine outcomes such as BMC. Our study adds a longitudinal perspective, even including comparisons with a reference population, and opens gates to trials evaluating the specific effects of resistance training on bone parameters.

The implications are substantial, particularly given the increased risk of fractures among individuals with type 1 diabetes (9). A systematic review and meta-analysis further support the notion that muscular fitness should be considered a critical marker of bone health during growth and development, given its strong association with BMC and BMD in healthy young populations (47). This study underscores the importance of incorporating muscular fitness as a central component of bone health assessment and intervention strategies.

The muscle-bone functional unit provides a plausible framework for interpreting these findings. According to Frost's mechanostat theory, the mechanical forces generated to increase muscular fitness during growth serve as critical stimuli for bone mass accrual, regulating the activity of osteoblasts and osteoclasts (48). This framework underscores the importance of muscular fitness in type 1 diabetes, a condition characterized by low bone turnover and, in particular, less bone formation (49). Additional explanations may arise from the indirect influence of muscular fitness on various factors implicated in the bone complications associated with type 1 diabetes (10). Improved muscular fitness could mitigate the adverse effects of glycemic dysregulation, chronic inflammation, and hormonal imbalances, promoting bone health. These insights further highlight the critical role of muscle-strengthening interventions as part of a comprehensive strategy to address bone health challenges in this population.

### Limitations and Strengths

This study sheds light on the relationship between muscular fitness and bone health in youth with type 1 diabetes, but several limitations warrant consideration. Bone health was assessed using DXA, as recommended by the American Diabetes Association for BMC and aBMD monitoring, but it lacks the ability to measure geometric or microarchitectural properties available via peripheral quantitative computed tomography (pQCT) (5, 6). DXA analyses were limited to whole body scan while the hip and lumbar spine region-specific analyses would have provided additional data of interest. In this study, lean mass was not included as a covariate due to multicollinearity with BMI, although BMI was used as a practical and predictive alternative as in previous studies (50). Vitamin D status, a potential factor in bone health (10), was omitted, although its influence remains debated (44). Additionally, we did not control the analyses by history of prior fractures, which could influence bone health and potentially introduce minor confounding. Finally, residual confounding cannot be ruled out despite adjusting for major confounders using the directed acyclic graph method.

Despite these limitations, this study has several strengths. The longitudinal design with 3 assessments provides a more complete understanding of the relationship between muscle fitness and bone health over time, addressing the limitations of cross-sectional studies. In addition, multiple dimensions of muscle fitness are assessed, emphasizing muscle power as a key factor rather than relying solely on grip strength. Moreover, a clinically contrastable approach with reference population is provided, reinforcing muscle strength as a predictive marker of bone health in young people with type 1 diabetes without the need for assessments involving the use of x-rays.

## Conclusion

This longitudinal study underlines the critical role of muscular fitness in promoting bone health in children and adolescents with type 1 diabetes. A higher handgrip strength and other muscular fitness parameters were strongly associated with increased BMC and aBMD across multiple skeletal regions. These findings were further validated using standardized reference data from the general population, providing robust support for the observed relationships.

The results underscore the importance of incorporating muscle-strengthening activities into routine diabetes care as a prevention strategy to mitigate the adverse effects of type 1 diabetes on bone health. However, given the nature of this study, causal inferences cannot be drawn. Therefore, future research should focus on elucidating the underlying biological mechanisms through rigorously designed randomized controlled trials evaluating the direct impact of muscle-strengthening interventions on a comprehensive set of bone outcomes.

## Data Availability

Some or all datasets generated during and/or analyzed during the current study are not publicly available but are available from the corresponding author on reasonable request.

## Abbreviations

aBMD: areal bone mineral density
BMC: bone mineral content
BMI: body mass index
DXA: dual-energy x-ray absorptiometry
HbA1c: glycosylated hemoglobin
m*g*: milli-gravity
PHV: peak height velocity
RM: repetition maximum
TBLH: total body less head
